# Bacteriophages Contribute to Shaping *Clostridioides (Clostridium) difficile* Species

**DOI:** 10.3389/fmicb.2018.02033

**Published:** 2018-08-31

**Authors:** Louis-Charles Fortier

**Affiliations:** Department of Microbiology and Infectious Diseases, Faculty of Medicine and Health Sciences, Université de Sherbrooke, Sherbrooke, QC, Canada

**Keywords:** *Clostridium difficile*, *Clostridioides difficile*, bacteriophages, prophages, toxins, virulence, lysogenic conversion

## Abstract

Bacteriophages (phages) are bacterial viruses that parasitize bacteria. They are highly prevalent in nature, with an estimated 10^31^ viral particles in the whole biosphere, and they outnumber bacteria by at least 10-fold. Hence, phages represent important drivers of bacterial evolution, although our knowledge of the role played by phages in the mammalian gut is still embryonic. Several pathogens owe their virulence to the integrated phages (prophages) they harbor, which encode diverse virulence factors such as toxins. *Clostridioides (Clostridium) difficile* is an important opportunistic pathogen and several phages infecting this species have been described over the last decade. However, their exact contribution to the biology and virulence of this pathogen remains elusive. Current data have shown that *C. difficile* phages can alter virulence-associated phenotypes, in particular toxin production, by interfering with bacterial regulatory circuits through crosstalk with phage proteins for example. One phage has also been found to encode a complete binary toxin locus. Multiple regulatory genes have also been identified in phage genomes, suggesting that their impact on the host can be complex and often subtle. In this minireview, the current state of knowledge, major findings, and pending questions regarding *C. difficile* phages will be presented. In addition, with the apparent role played by phages in the success of fecal microbiota transplantation and the perspective of phage therapy for treatment of recurrent *C. difficile* infection, it has become even more crucial to understand what *C. difficile* phages do in the gut, how they impact their host, and how they influence the epidemiology and evolution of this clinically important pathogen.

## Bacteriophages

**FIGURE 1:**
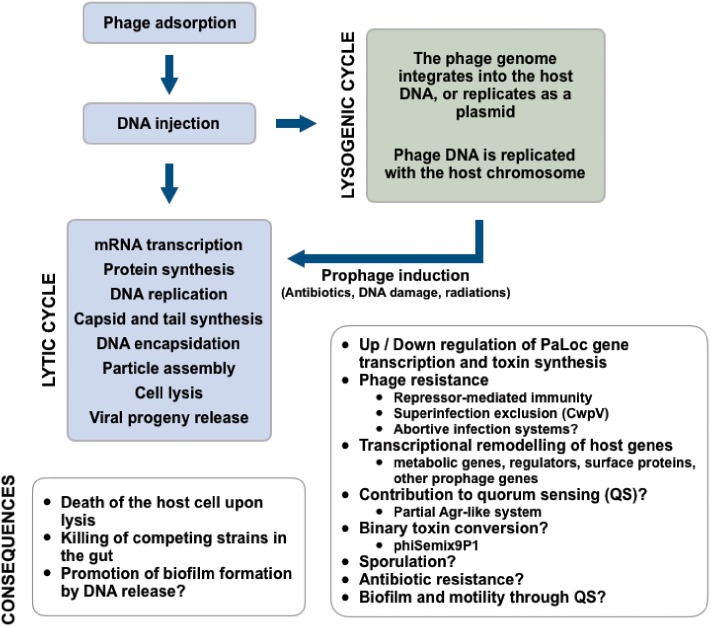
Summary of the replication strategies and multiple impacts of phages infecting *C. difficile*.

Bacteriophages (phages) are bacterial viruses that infect bacteria. At ∼10^31^ viral particles, they represent the most abundant biological entities in the biosphere and almost all bacteria are susceptible to phage attacks. Phages are therefore important drivers of bacterial evolution (Brüssow and Hendrix, 2002). Depending on the phages’ replication strategy, their impact on bacterial populations can be drastically different. The two most frequent mechanisms of phage replication are the lytic cycle, and the lysogenic cycle (**Figure 1**). Phages that replicate only via the lytic pathway are referred to as “**virulent**” and inevitably lead to death of the host upon infection. Those phages that can replicate either by the lytic or the lysogenic cycle are said to be “**temperate**.” When temperate phages become integrated into the host genome (i.e., prophages) their host is said to be lysogenic.

## Bacteriophages Infecting *C. Difficile*

Most phages infecting *Clostridioides (Clostridium) difficile* were isolated following induction of prophages (Shan et al., 2012; Hargreaves and Clokie, 2014; Sekulovic et al., 2014). However, free phages have also been isolated directly from fecal supernatants of patients infected with *C. difficile*, hence prophage induction occurs *in vivo* (Meessen-Pinard et al., 2012). In the 1980s and 1990s, *C. difficile* phages were studied for their potential as strain typing tools (Sell et al., 1983; Dei, 1989; Mahony et al., 1991). Now their potential for phage therapy applications is being explored (Nale et al., 2016b, 2018). At the time of writing this manuscript, at least 24 complete phage genomes were available in public databases (**Table 1**). Most of them are members of the *Myoviridae* family of the order *Caudovirales* (i.e., phages with contractile tails), and six phages are members of the *Siphoviridae* family (i.e., phages with long non-contractile tails) (Ackermann and Prangishvili, 2012). Functional data describing the lytic cycle of *C. difficile* phages are quite scarce. The few one-step growth curve experiments published so far suggest highly variable latency periods (from ∼30 min to 2 h), (Goh et al., 2005b; Sekulovic et al., 2011) and burst sizes (i.e., virions released per infected cell), with as few as 5 phages/cell for phage ϕC2 (Goh et al., 2005b), to 122 phages/cell for phage 56 (Mahony et al., 1985). In addition, most phages have relatively narrow host ranges (Goh et al., 2005b; Sekulovic et al., 2011, 2014; Rashid et al., 2016), which is directly related to the availability of a suitable host receptor, the presence of endogenous prophages conferring resistance, and the presence of antiphage systems including clustered regularly interspaced short palindromic repeat sequences (CRISPRs) (Hargreaves et al., 2014a; Boudry et al., 2015), the recently described superinfection exclusion system CwpV (Sekulovic et al., 2015), and possibly others. Of particular interest, the receptor(s) used by *C. difficile* phages to infect their host remain(s) to be clearly identified. In other Gram-positive bacteria, different cell surface components are used as phage receptors, from single proteins to polysaccharides or teichoic acids. As examples, the *Bacillus subtilis* YueB (São-José et al., 2006) and *Lactococcus lactis* Pip (Babu et al., 1995) proteins are, respectively, used by phages SPP1 and c2 to infect their host. Different polysaccharides composing the pellicle are used as receptors by lactococcal phages such as p2 (Bebeacua et al., 2013). Of note, recent data with Diffocins, i.e., phage tail-like bacteriocins that resemble *Myoviridae* phage tails and that kill their host by puncturing the cell, point to a central role of the surface layer protein A (SlpA) as a general phage receptor used by Diffocins and *Myoviridae* phages (Gebhart et al., 2015; Kirk et al., 2017). Detailed molecular interactions between phages and the *C. difficile* surface need to be further investigated, in particular regarding *Siphoviridae* phages, and considering the potential of phages as therapeutic agents.

**Table 1:** List and relevant characteristics of *C. difficile* phages for which a complete genome sequence is available in GenBank.

Phage	Family^∗^	Genome size (bp)	Accession No.	Date of Release	Relevant characteristics/observations	Reference
phiCD119	M	53,325	AY855346.1	2006	• First *C. difficile* phage genome to be sequenced • The phage repressor RepR was shown to repress transcription of the five PaLoc genes through binding to the *tcdR* promoter element	Govind et al., 2006, 2009
phiC2	M	56,538	DQ466086.1	2007	• Increased TcdB production in certain lysogens carrying phiC2, although transcription of the *tcdB* gene remained unaffected • Transcription of the *tcdA* gene was increased or reduced in some lysogens, although the level of TcdA toxin remained unaffected •phiC2 was shown to promote transduction of erythromycin resistance through transfer of the *erm*(B) gene carried on Tn*6215*	Goh et al., 2005a, 2007, 2013
phiCD27	M	50,930	NC_011398.1	2008	• The phage’s endolysin gene was cloned and expressed in *Escherichia coli* and *Lactococcus lactis*. The specificity of the lytic activity of the CD27L endolysin toward *C. difficile* was demonstrated using a panel of 30 *C. difficile* isolates + other commensal bacteria • phiCD27 significantly reduced the burden of *C. difficile* and toxin production in prophylactic assays using *in vitro* batch fermentation and human colon models	Mayer et al., 2008; Meader et al., 2010, 2013
phiCD6356	S	37,664	NC_015262.1	2010	• First *C. difficile* *Siphoviridae* phage genome to be sequenced	Horgan et al., 2010
phiCD38-2	S	41,090	HM568888.1	2011	• Increased toxin production and PaLoc gene transcription in ribotype 027 lysogens carrying the phiCD38-2 prophage. The impact of phiCD38-2 on toxin production was strain-dependent. • First demonstration of a *C. difficile* prophage genome maintained as an extrachromosomal plasmid • RNAseq analysis of a R20291-lysogen carrying phiCD38-2 led to the discovery of the antiphage activity of the CwpV phase-variable surface protein	Sekulovic et al., 2011, 2015
phiMMP02 phiMMP04	M M	48,396 31,674	JX145341.1 JX145342.1	2012 2012	• Free phage particles were isolated from filter-sterilized fecal supernatants from *C. difficile* infected patients, demonstrating that prophage induction occurs *in vivo*, during infection	Meessen-Pinard et al., 2012; Boudry et al., 2015
phiCDHM1	M	54,279	NC_024144.1	2013	• The phage genome encodes three homologs of the *Staphylococcus aureus* Agr quorum sensing (QS) system, namely *agrD* (pre-peptide of an autoinducing peptide, AIP), *agrB* (processes the pre-AIP), and *agrC* (histidine kinase) • Possibly affects QS-mediated phenotypes, although no AgrA-like response regulator could be identified in phiCDHM1 • A cocktail containing phiCDHM1, phiCDHM2, phiCDHM5, and phiCDHM6 was shown to reduce *C. difficile* burden *in vitro* and in colonization experiments in hamsters and in a *Galleria mellonella* larva *C. difficile* infection model. The phage cocktail also prevented biofilm formation *in vitro*	Hargreaves et al., 2014b; Nale et al., 2016a,b
phiCDHM13	M	33,596	HG796225.1	2013		Hargreaves et al., 2014a
phiCDHM14	M	32,651	LK985321.1	2014		Hargreaves et al., 2014a
phiCDHM19	M	54,295	LK985322.1	2014		Hargreaves et al., 2014a
phiCD211 phiCDIF1296T	S S	131,326 131,326	NC_029048.1 CP011970.1	2014 2015	• phiCD211 and phiCDIF1296T are identical phages • Large phage genome maintained as an extrachromosomal plasmid in lysogens • The genome encodes several genes potentially affecting various phenotypes in *C. difficile*, including antimicrobial resistance, spore germination, and CRISPR-mediated resistance • Encodes a CRISPR-*cas3* gene in addition to a CRISPR array	Boudry et al., 2015; Wittmann et al., 2015; Garneau et al., 2018
phiCD24-1	S	44,129	LN681534.1	2015		Boudry et al., 2015
phiCD111	S	41,560	LN681535.1	2015		Sekulovic et al., 2014; Boudry et al., 2015
phiCD146	S	41,507	LN681536.1	2015		Sekulovic et al., 2014; Boudry et al., 2015
phiMMP01 phiMMP03	M M	44,461 52,261	LN681541.1 LN681542.1	2015 2015	• Free phage particles were isolated from filter-sterilized fecal supernatants from *C. difficile* infected patients, demonstrating that prophage induction occurs *in vivo*, during infection	Meessen-Pinard et al., 2012; Boudry et al., 2015
phiCD481-1	M	32,846	LN681538.1	2015		Sekulovic et al., 2014; Boudry et al., 2015
phiCD505	M	49,316	LN681539.1	2015		Sekulovic et al., 2014; Boudry et al., 2015
phiCD506	M	33,274	LN681540.1	2015		Sekulovic et al., 2014; Boudry et al., 2015
phiCDHM11	M	32,000	HG798901.1	2015		Hargreaves and Clokie, 2015
phiCDKM9	M	49,822	KX228399	2016		Rashid et al., 2016
phiCDKM15	M	50,605	KX228400	2016		Rashid et al., 2016
phiSemix9P1	N∖ A	56,606	KX905163.1	2017	• The phage genome encodes a complete and functional binary toxin locus (CdtLoc)	Riedel et al., 2017

*^∗^M, Myoviridae; S, Siphoviridae*.

## High Prevalence of Prophages in *C. Difficile* Genomes

Over 1,300 *C. difficile* genomes have been fully sequenced and are available in public repositories, but thousands of additional genomes have also been sequenced and are available through collaborative research (Garneau et al., 2018). Although uncommon, as many as 5–6 different prophages were identified in a single *C. difficile* genome (Amy et al., 2018; Ramírez-Vargas et al., 2018). However, between 1 and 3 prophages are more frequently observed, in addition to genomic “islands” containing phage-related genes. Recent studies highlighted the prevalence of large phage genomes that reside as extrachromosomal DNA in *C. difficile* (Garneau et al., 2018; Ramírez-Vargas et al., 2018). For example, the large phiCD211/phiCDIF1297T and related phages, with genomes of ≥131-kb, have been detected in 5% of 2,584 *C. difficile* genomes analyzed, spanning 21 different multi-locus sequence types (MLST) (Wittmann et al., 2015; Garneau et al., 2018). Ten other large phage genomes (∼128–135-kb), including phiCD5763, phiCD5774, and phiCD2955, were recently described in *C. difficile* isolates from around the world and representing seven different MLST sequence types (Ramírez-Vargas et al., 2018). Comparative genomic analyses underlined the important genetic variability among large phages, and they could eventually be used as genetic markers to subtype and monitor specific strains during epidemiological studies, as suggested for *Salmonella enterica* (Mottawea et al., 2018).

It is worth mentioning that extrachromosomal phage genomes can be difficult to differentiate from large plasmids containing phage genes. A study by Amy et al. (2018) reported the characterization of a large plasmid in *C. difficile* strain DLL3026. This 46-kb plasmid, called pDLL3026, and several other plasmids of similar size identified in other isolates, harbor a significant number of phage structural genes coding for head and tail morphogenesis, recombinases/integrases and phage regulators. The presence of partition genes like *parM* and *parR* and DNA similarity with plasmids led the authors to conclude that these were plasmids. However, the presence of partition genes like *parA* has also been reported in other phages, including phiCD6356 (Horgan et al., 2010), ϕCD38-2 (Sekulovic et al., 2011), and phiSemix9P1 (Riedel et al., 2017), the latter two known to be maintained as extrachromosomal DNAs in lysogenic cells. Large phage genomes such as phiCD211/phiCDIF1296T and phiCD5763 also seem to be frequently found as extrachromosomal DNA, and ParM homologs were identified in some of them (Garneau et al., 2018; Ramírez-Vargas et al., 2018). Therefore, in the absence of functional data to assess the inducibility and production of infectious particles from these large “plasmids,” it is hard to conclude on their exact nature.

The identification of complete prophages in bacterial genomes has been greatly improved, thanks to the development of tools such as PHAST and PHASTER (Arndt et al., 2016, 2017). But the task is more challenging with decaying prophage remnants that have lost many of the conserved phage components such as structural genes. Yet, these remnants could still influence their host even if they can’t replicate or produce complete infectious particles. Diffocins are a good example: these phage tail-like particles resemble *Myoviridae* phage tails, but lack a capsid and genetic material (Gebhart et al., 2012). They kill their host following induction and lysis of the cell, and also kill other competing cells around, but they can’t produce infectious particles. The functional role of Diffocins remains to be clarified, but they possibly provide a competitive advantage to *C. difficile* strains carrying them by killing surrounding competitors (Kirk et al., 2017).

## The Consequences of Prophage Induction

The role of prophages in the physiology and virulence of *C. difficile* is a topic of great interest, considering their prevalence and diversity, and the historical role prophages played in the virulence of other bacterial pathogens (Brüssow et al., 2004; Fortier and Sekulovic, 2013). Prophage stability is critical because of the direct consequence on the viability of the host itself and susceptible surrounding strains/species that can be re-infected. Induction can occur spontaneously, but is promoted by common antibiotics and various environmental stresses (Rokney et al., 2008; Meessen-Pinard et al., 2012; Shan et al., 2012). Of note, prophage induction triggered by antibiotics promote horizontal gene transfer and spreading of antibiotic resistance genes in mice (Modi et al., 2013). *In vitro*, phage ϕC2 was shown to mediate transduction of the Tn*6215* transposon between *C. difficile* strains (Goh et al., 2013). Differences in abundance and diversity of gut phages has also been associated with diseases and could be the result of prophage induction (Norman et al., 2015; Manrique et al., 2016). Hence, better understanding the role of prophage induction in complex ecosystems such as the gut is of great interest.

Induction of prophages and phage-related elements can also have other important physiological roles in *C. difficile*. For example, excision of the phage-related mobile element called *skin^Cd^* is important during the sporulation process (Haraldsen and Sonenshein, 2003; Saujet et al., 2014). The *skin^Cd^* is a putative prophage remnant similar to the one identified in *B. subtilis* (*skin^Bs^*) that interrupts the coding sequence of SigK, a sporulation-associated alternative sigma factor. Excision of the *skin^Cd^* element at a specific time point during the sporulation process restores the coding sequence of the gene, allowing expression of *sigK* (Saujet et al., 2014; Fimlaid and Shen, 2015). Control of the excision of the *skin* element remains unclear, but a putative site-specific recombinase similar to SpoIVCA, encoded by *cd1231* and located within *skin^Cd^*, is suspected to be involved. Some crosstalk between prophages has been reported (Lemire et al., 2011; Sekulovic and Fortier, 2015), including between their recombinases (Singh et al., 2014). Therefore, other phage-encoded recombinases could possibly participate in *skin^Cd^* excision as well, and thus influence sporulation (Saujet et al., 2014).

## Prophages Influence Toxin Production in *C. Difficile*

The main virulence factors of *C. difficile* are the large TcdA and TcdB exotoxins. They are encoded on a 19.6-kb pathogenicity locus, the PaLoc (Rupnik et al., 2009). The PaLoc is thought to originate from an ancient prophage, since it shares a number of features with phages, in particular the *tcdE* gene encoding a phage-like holin involved in toxin secretion (Govind and Dupuy, 2012; Govind et al., 2015; Monot et al., 2015). Prophage induction *per se* has not been directly associated with toxin release or synthesis in *C. difficile*, as opposed to Shiga toxin-encoding phages in *Escherichia coli* (Kimmitt et al., 1999; Zhang et al., 2000) but some prophages interfere with toxin synthesis. For example, phage ϕCD119 was shown to express the RepR repressor, capable to bind a DNA region in the promoter of *tcdR* in the PaLoc, resulting in repression of toxin genes (Govind et al., 2009). On the contrary, phage ϕCD38-2 was shown to increase transcription of all five PaLoc genes by a yet unknown mechanism, resulting in more toxins produced *in vitro*. However, the impact of ϕCD38-2 on toxin synthesis was strain-dependent (Sekulovic et al., 2011) and similar observations were reported with other *C. difficile* phages (Goh et al., 2005a), suggesting that the influence of a prophage on its host partly depends on the genetic background.

A complete binary toxin locus (CdtLoc) has been recently identified in the genome of phage phiSemix9P1 (Riedel et al., 2017). The binary toxin, normally located on a 6.2-kb chromosomal locus called CdtLoc comprises 2 genes coding for the toxin components, *cdtA* and *cdtB*, as well as a regulator encoded by *cdtR* (Carman et al., 2011; Gerding et al., 2014). Of note, all three genes from the CdtLoc were shown to be transcribed from the phiSemix9P1 prophage, suggesting that it is functional, although no toxin assays have been performed (Riedel et al., 2017). The CdtLoc is present only in a subset of *C. difficile* isolates, including the epidemic ribotype 027 isolates (Bauer et al., 2011), and studies suggest that CDT contributes to virulence by promoting adhesion to epithelial cells (Schwan et al., 2009, 2014; Gerding et al., 2014). phiSemix9P1 has limited DNA homology with another *C. difficile* phage, ϕCD505, and the large pCDBI1 plasmid, suggesting that it is genetically unique. The identification of a CDT-encoding phage is intriguing but it might represent a rare isolated case, since it has never been observed in other *C. difficile* phages, including the numerous prophages identified in the course of genome sequencing projects. Nevertheless, it further supports the evolutionary role of phages in toxin conversion of *C. difficile* (Riedel et al., 2017).

## Prophage Gene Expression During Lysogeny

During active phage replication, the transcriptional program of the host is profoundly restructured and metabolic resources are redirected toward phage replication. However, during the lysogenic cycle, prophages are generally quiescent and minimal gene transcription is observed from the prophage itself. Only a few gene products are required to establish and maintain lysogeny (Ainsworth et al., 2013), the CI repressor from the *E. coli* phage Lambda being the most well-characterized gene expressed during lysogeny (Oppenheim et al., 2005; Rokney et al., 2008).

Very little is known about transcriptional reprogramming during phage infection or lysogeny in *C. difficile*. In fact, only one study has looked at global gene expression during lysogeny (Sekulovic and Fortier, 2015). In that study, the ϕCD38-2 prophage was introduced into the epidemic strain R20291 and mRNA levels were assessed by RNAseq. It is important to mention that the prophage was maintained as a circular extrachromosomal DNA, so the host genome integrity was unaffected. On a genome-wide scale, the expression of 39 genes was significantly altered by the introduction of the prophage, including genes from the phi027 prophage already present in the host. This further supports the existence of some crosstalk between prophages (Sekulovic and Fortier, 2015). Two-thirds of the differentially expressed genes were downregulated twofold to threefold, and half of the differentially expressed genes were related to sugar uptake and metabolism, suggesting a possible impact on growth kinetics. Of note, the *cwpV* gene encoding a conserved surface protein was induced 20-fold in the lysogen. Transcription of *cwpV* is dependent on the configuration of a genetic switch located between the promoter and the gene. Recombination of the switch, catalyzed by the host-encoded RecV recombinase, turns transcription of *cwpV* ON or OFF in a phase-variable manner (Reynolds et al., 2011; Fagan and Fairweather, 2014). Only ∼5% of bacterial cells in culture express the CwpV protein at their surface, but in the R20291 lysogen carrying ϕCD38-2, this proportion increased to 95%, hence explaining the higher mRNA levels observed. The exact mechanism by which ϕCD38-2 influences phase variation remains unknown (Sekulovic and Fortier, 2015). CwpV is a large conserved cell wall protein suspected to contribute to cell adhesion and biofilm formation, and possibly immune evasion (Reynolds et al., 2011). The location of the protein at the cell surface and its apparent link with lysogeny suggested that it could play some role in phage infection. It turned out that CwpV has strong antiphage activity against several *C. difficile* phages of the *Siphoviridae* and *Myoviridae* families when overexpressed from a plasmid or from a “locked-ON” strain. Current data suggest that CwpV functions as a superinfection exclusion system (Sekulovic et al., 2015) that blocks phage DNA injection. The biological relevance of such an antiphage system seems obvious in the context of the gut microbiota. CwpV-ON strains would be protected from lytic phage attacks, which are expected to be relatively frequent in the gut due to high phage and bacterial densities (Manrique et al., 2016, 2017). Higher numbers of CwpV-ON cells could also contribute to colonization of the gut through increased bacterial adhesion and biofilm formation. Maybe of greater concern, however, is the fact that *cwpV*-expressing cells are naturally occurring *in vitro* due to phase variation and these cells are resistant to phage infection. Hence, looking at future phage therapy perspectives (Nale et al., 2016b), naturally occurring CwpV-positive cells in the gut could potentially compromise the efficacy of therapeutic phages. Further *in vivo* assays will be required to clarify the biological role and consequences of CwpV expression.

## Other Impacts of Prophages on their Host

Several phage genomes carry cargo genes unrelated to the phage replication cycle, and their expression is often independent from the phage circuitry and occurs during lysogeny. The genes often code for virulence factors, including toxins, superantigens, and hydrolytic enzymes (Brüssow et al., 2004; Fortier and Sekulovic, 2013). Certain prophage genes can also provide phage immunity via superinfection exclusion (Mahony et al., 2008; Labrie et al., 2010).

The genomes of many *C. difficile* prophages encode genes that are suspected to influence their host. For example, the large phiCD211-like phages encode putative multidrug resistance genes, spore proteases, and multiple regulators that could interfere with host regulation (Garneau et al., 2018). A CRISPR array with a *cas3* gene was also identified, suggesting that phiCD211-like phages possibly participate in CRISPR interference. The presence of CRISPR arrays has been reported in other *C. difficile* phages, including the two prophages from strain 630 (Hargreaves et al., 2014a; Boudry et al., 2015) as well as the phi027 prophage present in the epidemic strain R20291 and most R027 isolates (Sekulovic and Fortier, 2015). Transcriptomic analyses by RNAseq showed that these CRISPR arrays are transcribed and thus, possibly contribute to *C. difficile* resistance to invading DNA (Boudry et al., 2015; Sekulovic and Fortier, 2015).

Phage phiCDHM1 and other predicted *C. difficile* prophages encode homologs of an Agr-like quorum sensing (QS) system (Hargreaves et al., 2014b). QS is used to coordinate specific phenotypes at the whole population level in function of cell density. QS has been implicated in virulence of several pathogens, by coordinating toxin secretion, biofilm production, motility, and sporulation (Novick and Geisinger, 2008; Antunes et al., 2010; Rutherford and Bassler, 2012). The Agr system of *Staphylococcus aureus* is the most well-characterized QS system in Gram-positive bacteria, and regulates the expression of hundreds of genes, including exotoxins and surface proteins. It is encoded by an operon of four genes, *agrD-agrB-agrC-agrA* (Novick and Geisinger, 2008). At least two types of QS systems have been described in *C. difficile*; one is similar to the *S. aureus* Agr, while the other is related to the luxS/AI-2 from *Vibrio harveyi* (Stabler et al., 2009). Both systems control the expression of *C. difficile* toxins in function of cell density (Lee and Song, 2005; Martin et al., 2013; Darkoh et al., 2015). During lysogeny, the *agrB* and *agrC* genes are expressed from the phiCDHM1 prophage, suggesting that these components of the QS system are active. However, in the absence of an *agrA* homolog in phiCDHM1, it is impossible to conclude if the system is functional or not and whether it participates in some way to QS. We can speculate that AgrB and AgrC contribute to autoinducer secretion and signal detection, but no response would be elicited due to the absence of an associated response regulator. Alternatively, these phage-encoded genes could partly complement another Agr system from the host (Hargreaves et al., 2014b). QS likely affects multiple phenotypes in *C. difficile* (Martin et al., 2013) so it will be interesting to establish whether phage-encoded QS genes influence virulence-associated phenotypes such as toxin production, sporulation, or biofilm formation. QS signals detected by the phage-encoded AgrC could also lead to prophage induction, as observed with soil bacteria (Ghosh et al., 2009). This could be a means for the prophage to “determine” the best moment to initiate a replication cycle that will ensure its successful propagation into the bacterial population. It is therefore reasonable to hypothesize that during infection of the gut, high cell densities would trigger prophage induction, hence promoting phage dissemination and possibly horizontal gene transfer. Further research on the impact of QS on prophage stability would be necessary.

## Conclusion and Perspectives

The contribution of phages to the evolution and virulence of *C. difficile* remains to be clarified (Fortier and Sekulovic, 2013). So far, prophages seem to impact *C. difficile’s* lifestyle and biology in subtle ways, depending on the genetic background of the host. Studying phage–host interactions requires extensive knowledge of the biology of the phage and the host. Unfortunately, many phage genes have no homologs in databases or have no assigned function. Therefore, one way to investigate the impact of a prophage on its host is to introduce a given temperate phage into a susceptible bacterial host to create a new lysogen and to study various phenotypes in comparison with the parental strain lacking that prophage. However, bacterial genomes often carry multiple prophages and phenotypes can sometimes result from the cumulative effects of more than one prophages, like reported for the DNAses secreted by *Streptococcus pyogenes* SF370 (Euler et al., 2016). In addition, natural lysogens have been carrying prophages for extensive periods of time and as such, the prophages’ regulatory circuits are often seamlessly integrated into the host network (Ehrbar and Hardt, 2005). Therefore, a better alternative is to remove parts or whole prophages from their natural lysogen to study their impact. Such “prophage-cured” strains can then be compared with the lysogenic parental strain. Curing lysogens from their prophages can be quite challenging depending on the host and the availability of molecular tools. Reports of successful curing using extensive screening for spontaneous prophage-cured mutants, or using allelic exchange with counter selection methods have been published in Gram-negative (e.g., *E. coli*) and Gram-positive bacteria (e.g., *S. pyogenes, S. aureus*). These studies have shed light on the role of individual prophages as well as their combined contribution to virulence of their host (Bae et al., 2006; Wang et al., 2010; Euler et al., 2016). Of note, curing of one of the two prophages from *C. difficile* strain 630 has been recently reported, and involved the use of the CRISPR technology (Hong et al., 2018). This first example of prophage curing in *C. difficile* paves the way for additional studies on the role of prophages in this pathogen, in particular in epidemic strains such as the R20291 that carries the conserved phi027 prophage (Stabler et al., 2009). Better understanding how phages interact with *C. difficile* at the molecular level will be essential, especially for future phage therapy applications. Hence research focusing on identifying the cell receptor(s) and the phages’ receptor binding protein and how these two influence the phages’ host range will be crucial. In addition, understanding how phages affect *C. difficile* and whole bacterial populations in complex ecosystems such as the gut microbiota will be determinant as well. For instance, transfer of certain phages from donors to recipients seems to contribute to the success of fecal microbiota transplantation to treat recurrent *C. difficile* infections (Zuo et al., 2017). Studying the interplay between the virome and the microbiome in health and disease is thus of high relevance. In conclusion, there is a lot more to discover about *C. difficile* phages and the newly developed molecular tools and the availability of bacterial genome sequences will certainly foster research in this domain.

## Author Contributions

L-CF collected the ideas, concepts, and interpretations, as well as wrote the manuscript.

## Conflict of Interest Statement

The author declares that the research was conducted in the absence of any commercial or financial relationships that could be construed as a potential conflict of interest.
